# Prevalence and Associated Symptoms of* Helicobacter pylori* Infection among Schoolchildren in Kassala State, East of Sudan

**DOI:** 10.1155/2018/4325752

**Published:** 2018-01-15

**Authors:** Mohammed Abbas, Faiza A. Sharif, Shamselfalah M. Osman, Abdallah M. Osman, Sulieman M. El Sanousi, Mamoun Magzoub, Mutasim E. Ibrahim

**Affiliations:** ^1^Department of Pediatrics, College of Medicine, University of Bisha, Bisha, Saudi Arabia; ^2^Department of Pediatrics, Faculty of Medicine & Health Sciences, University of Kassala, Kassala, Sudan; ^3^Department of Microbiology, Faculty of Medicine & Health Sciences, University of Kassala, Kassala, Sudan; ^4^Department of Pediatrics, King Saud Medical City, Riyadh, Saudi Arabia; ^5^Department of Microbiology, Faculty of Veterinary Medicine, University of Khartoum, Khartoum, Sudan; ^6^Department of Parasitology, Faculty of Medical Laboratory Science, International University of Africa, Khartoum, Sudan; ^7^Department of Clinical Microbiology and Parasitology, College of Medicine, University of Bisha, Bisha, Saudi Arabia

## Abstract

This study aimed to determine the prevalence of* H. pylori* infections among schoolchildren and investigate the associations between* H. pylori* seropositivity and existence of gastrointestinal symptoms.* Methods*. A prospective cross-sectional study was conducted during a period from January to December 2012 at Kassala state, east of Sudan. Schoolchildren from different primary schools were enrolled in the study. Sociodemographic characteristics and gastrointestinal symptoms were recorded from each child. A rapid immunochromatographic test was performed for the detection of* H. pylori* IgG antibodies. Data on patient demographic characteristics, clinical diagnosis, and findings of* H. pylori* infection were analyzed by simple descriptive statistics.* Results*. Among 431 schoolchildren,* H. pylori* seropositivity was found to be 21.8%. The majority of children (79; 84%) had BMI below the normal range. The most frequent symptoms associated with* H. pylori* infections were nausea (25.5%), followed by gastric pain (24.5%) and heart pain (20.2%). There were statistically significant differences in* H. pylori* seropositivity between boys and girls (*p* = 0.003).* Conclusions*. The prevalence of* H. pylori* infection among schoolchildren in Kassala city has been documented. Although the majority of the disease was associated with several gastrointestinal symptoms, the role of infection in the etiology of abdominal symptoms needs further investigations.

## 1. Introduction

*Helicobacter pylori (H. pylori)* is a gram-negative bacterium colonizing human stomach and associated with numerous gastrointestinal diseases [1]. It is quite a frequent infection all over the world; more than half of the population in both developed and developing countries are infected with this microorganism [2, 3]. Most of the people acquire* H. pylori* infection during their early childhood [4].* H. pylori* has been reported as a common cause of chronic gastritis, peptic ulcer diseases, and gastric cancer in adults [5].

Most of* H. pylori* infections usually are symptomless and without clinical manifestation, particularly in poor communities [6]. However, signs and symptoms associated with the disease are primarily due to gastric or peptic ulcer illness or duodenal inflammation. Furthermore, other symptoms such as nausea, vomiting, and abdominal pain may be attributed to other gastrointestinal diseases [7].

*H. pylori* infection developed during early childhood is considered as a significant risk factor for gastric carcinoma in the adult individual [8]. It is well known that* H. pylori* infection among children is associated with several extragastric diseases, including growth reduction, iron-deficiency anemia, and idiopathic thrombocytopenic purpura [1, 7]. Schoolchildren in developing countries are at-risk group due to several factors including low socioeconomic status, poor quality of drinking water, overcrowding, poor personal and environmental hygiene, and food contamination [2, 9].

Screening for the serum IgG antibody to* H. pylori* is a practical method for diagnosing* H. pylori* infection in children. However, measurements of the* H. pylori* IgG antibody are useful for monitoring treatment of* H. pylori* infection because of its high sensitivity and ease of performance [10, 11]. Therefore, this study aimed to determine the prevalence of* H. pylori* infections among schoolchildren in Kassala and to investigate the associations between* H. pylori* seropositivity and presence of gastrointestinal symptoms.

## 2. Materials and Methods

A prospective randomized cross-sectional study was conducted during a period from January to December 2012 at Kassala state, east of Sudan (about 500 kilometers from Khartoum capital). Kassala has a population of 180.000 of different ethnicity and Sudanese tribes [12]. The study population was comprised of schoolchildren, those who were selected from various primary schools in Kassala city.

### 2.1. Samples and Data Collection

A structured questionnaire has been developed to obtain sociodemographic characteristics and gastrointestinal symptoms. Filling of each questionnaire form was achieved by the authors (pediatricians) during their conduction of clinical examinations. Moreover, the body mass index (BMI) was obtained (weight in kg/height in m2) and the schoolchildren were classified as being underweight, healthy, and overweight. Then about 5 ml of blood sample was collected from each child in a plain container for detection of* H. pylori* antibodies. The collected samples were transported to the laboratory, and then sera were separated by centrifugation at 15.000 rpm for 10 minutes and stored at −20°C until being used for serological tests. Children under antibiotics treatment for the last four weeks, with the illness of the liver, renal, pancreatic, or parasitic infections, or evidence of any other viral or bacterial infections were excluded from the study. The study was approved by the ethical clearance committee, Ministry of Health, Kassala state, Sudan. Written consent was obtained from each participant or their parents or guardians after informing them about the importance of the study.

### 2.2. Screening of* H. pylori* IgG Antibodies

The IgG anti-*H*.* pylori* antibody in serum was detected using a Hexagon* H. pylori* commercial immunochromatographic test kit (HUMAN Gesellschaft für Biochemica und Diagnostica mbH, Germany) with a high sensitivity (96%) and specify (99%) as per manufacturer's specification. The procedure followed the manufacturer's instructions. In brief, 25 *μ*L of serum sample was added to the sample well of the strep using automatic pipette followed by adding 3 drops of buffer. The result was read and interpreted macroscopically after 15 minutes. Positive and negative controls were run simultaneously. Positive results were specified by two pink/red bands (control line and test line), seen in the result window of the test cassette, while a negative result was interpreted when only one pink/red band was observed in the control window. In case of no pink/red color developed in control and test line, the test cassette was invalid and the sample under analysis was repeated.

### 2.3. Data Analysis

Statistical Package for Social Sciences program (SPSS Inc., Chicago, IL, USA) version 16 was used for data entry and for analysis of the patients demographic characteristics; clinical diagnosis and findings of* H. pylori* infection were analyzed by simple descriptive statistics. Chi-square test was used to compare every two variables. A *p* value lower than 0.05 was statistically significant.

## 3. Results

A total of 431 children (217 boys and 214 girls) from different primary schools were enrolled in the study. The ages of the schoolchildren ranged from 6 to 18 years (mean 11.78 years + 2.035 (SD)).

*H. pylori* seropositivity was found to be 21.8% (94/431). The majority of the infected children (84%; 79/94) had BMI below the normal range. Of the 94 positive cases, most of them were boys 60 (27.7%), whereas 34 (15.9%) were girls. There were statistically significant differences in* H. pylori* seropositivity between boys and girls (*p* = 0.003) (Table 1).

**Figure 1:**
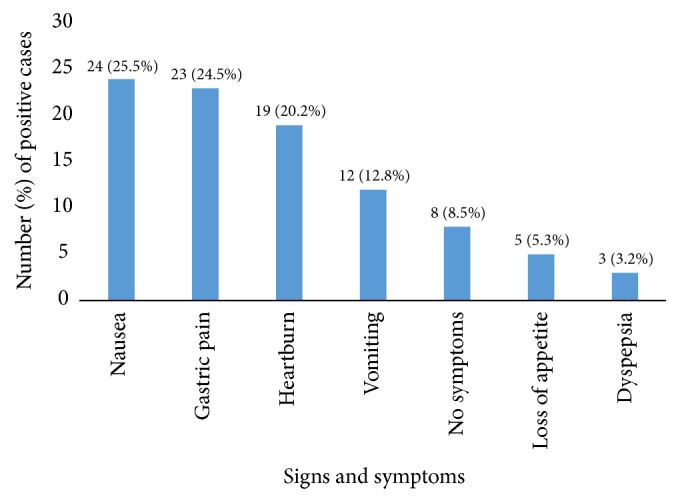
Frequency of symptoms associated with* H. pylori* infection among schoolchildren.

As shown in Figure 1, the most common symptoms associated with* H. pylori* infections were nausea (25.5%), followed by gastric pain (24.5%) and heart pain (20.2%).

There was no significant difference of* H. pylori* seropositivity between the all age groups of children (Table 2).

## 4. Discussion

Eradication of* H. pylori* infection has currently become an important concern because it can cause many gastroduodenal disorders [13]. To our best knowledge, this is the first study applying rapid one-step immune-chromatographic assay to screen the presence of* H. pylori* antibody among children in Kassala city, east of Sudan. In the present study, the prevalence of* H. pylori* among schoolchildren was 21.8%. This value is similar to those reported in Iran (19.8%) [13], but higher than 15.1% reported in Taiwan [8]. However, our finding was lower than 27.4% reported in Saudi Arabia [14] and 49% in Turkey [15]. Reports presented in this study, together with reports of others, indicate that there is quite a variation in prevalence of* H. pylori* infections all around the world. Tsongo et al. mentioned that the difference in results is probably due to the variations in the study population, such as the urban dwellers, and the age and health conditions of the patients [9]. Studies document that low socioeconomic status, sanitary conditions, level of educational background, and rates of immigrant children from the surrounding cities are significant risk factors for* H. pylori* infection among children [8, 16, 17]. Therefore, a proper approach to improve lifestyle, sanitary facilities, and promoting economic and educational status has an impact on the decline in the incidence of* H pylori* infections [17, 18]. However, prevention and eradication of* H. pylori* infection by treatment of children have been recommended [19]. The North American and European societies of Pediatric Gastroenterology, Hepatology, and Nutrition guidelines for the management of* H. pylori* infection in children recommend triple therapy as a first-line eradication regimen [20]. This treatment regimen should be with three medications including a proton pump inhibitor combined with amoxicillin and clarithromycin or metronidazole. The guidelines also recommended bismuth salts plus amoxicillin and metronidazole as a second-line therapy. Triple therapy should be administered for 7 to 14 days [19, 20]. However, development of antibiotic resistance strains, patient compliance, reinfection, and environmental factors might lead to treatment failure [18–20]. Therefore, understanding the epidemiologic burden of* H. pylori* infection is essential for implementing eradication strategies [18].

In the present study, the incidence of* H. pylori* infection was common among all age groups of schoolchildren. Worldwide studies determined the prevalence of* H. pylori* among school-aged children. For example, in Yemen, the prevalence of* H. pylori* in the age groups 6–8 and 9–12 years was higher than the other age groups [21]. In Saudi Arabia, the prevalence of infection was higher among students in intermediate school (43.7%) than those in secondary school (19.4%) [14]. A previous study on epidemiologic characteristics of* H. pylori* infection among different age groups of schoolchildren reported that the seropositive rates were 11.0% in 9–12 years age group and 12.3% in 13–15 years age group. Additionally, the prevalence of infection was increased by the age [8]. These findings indicated that school-aged children are the most common infected group with* H. pylori* infection with varying frequency. Therefore, establishing of health awareness program at school to enhance children personal hygiene and behavior might decrease the risk of disease.

In the present study, boys were more likely to have* H. pylori* infection in comparison to the girls. This finding in consistent with a survey carried out in Uganda [22]. Likewise, in Brazil, the acquisition of* H. pylori* infection was associated with the male gender [23]. In contrary to these findings, a study carried out in Yemen found that girls were more affected than boys [21]. However, elsewhere studies showed no differences in the prevalence of infection between boys and girls [15, 17].

*H. pylori* infection among children can be either clinically silent or associated with nonspecific sign or symptoms, which are seen in various childhood complaints [24]. In this study, the majority of* H. pylori* infection was associated with nausea (25.5%) and gastric pain (24.5%). Chong et al. suggested that* H. pylori* infection is more frequently associated with gastritis than with peptic ulcer disease in children and that* H. pylori* gastritis is a cause of recurrent abdominal pain syndrome in children [10]. However, the role of* H. pylori *infection in the etiology of abdominal symptoms remains unclear [25]. Furthermore, a recent study found that* H. pylori* infection is declining in symptomatic children, but it is still a common cause of upper gastrointestinal tract symptoms such as chronic abdominal pain/distress, epigastric pain, nausea, or vomiting [17].

*H. pylori* infection acquired in early childhood might affect growth and appetite, consequently influencing body weight of children [26, 27]. In the study, most of the positive cases were underweight. A study suggested that* H. pylori* infection hurts the growth of children [5]. Additionally, other authors have noted that a positive association of* H. pylori* infection with BMI was only seen in those aged 15+ years [4]. Recently, Benson et al. determined that the odds of being thin in the 10- to 19-year-old age group were 4.28-fold higher (95% CI 1.48–12.4) if they were* H. pylori* positive compared with those who were* H. pylori* negative [28]. In contrary,* H. pylori* infection was not associated with overweight/obesity observed from the retrospective study in this Chinese population [29]. Therefore, estimation of factors related to social and family environment may clarify the association between* H. pylori* infections and children's growth [4]. However, several factors can affect children's growth and stature, including nutrition, chronic inflammation, and gastrointestinal diseases [30, 31].

In conclusion, the prevalence of* H. pylori* infection among schoolchildren in this study was 21, 8%. We think this was a high infection among these children in the study area. Therefore, prevention and eradication of* H. pylori* infection by treatment of children might be essential. In addition, establishing of health awareness program at school to enhance children personal hygiene and behavior is essential to decrease the risk of infection. Although the majority of* H. pylori* infection is associated with different types of gastrointestinal symptoms, the role of* H. pylori* infection in the etiology of abdominal symptoms remains unclear, thus needing further investigations.

## Figures and Tables

**Table 1:** Gender-dependent frequency of *H. pylori* infection among schoolchildren (aged between 6 and 18 years).

Gender	Total number	Number (%) of positive cases	*p* value
Boy	217	60 (27.7)	0.003
Girl	214	34 (15.9)	

**Table 2:** Distribution of *H. pylori* infection among school-aged children according to their age groups at Kassala city, Sudan.

Age group	Total number of cases (*n* = 431)	Number of positive cases (*n* = 94)	% of positive cases
Less than 10 years	53	12	22.6
10 to 12 years	228	48	21.1
13 to 15 years	137	31	22.6
More the 15 years	13	3	23.1
